# Influence of marital pressures and cultural constructs on females (IMPACT-F): a global study of female medical students and doctors

**DOI:** 10.1186/s12909-025-08519-3

**Published:** 2026-01-03

**Authors:** Umme Summaiya Faisal, Ahmedyar Hasan, Sally Shayeb, Azeezat Oyewande, Majid Omari, Nino Dekanoidze, Awranoos Ahadi, Maryam Asif, Aron Shrestha, Sharanya Ezhilarasan Santhi, Umme Habiba Faisal, Kamal Kishan Akash, Norma Nicole Gamarra Valverde, Israa Ahmed Qutob, Naiela Ennaji Almansouri, Darja Golubeva, Ashmita Yadav, Mikias Lewoyehu Wondie, Zabi Fatima, Jaiprakash Suresh Gurav, Reshon Hadmon, Romana Riyaz, Sameer Asim Khan, Roopali Dahiya, Alen Sam Saji, Christos Tsagkaris, Ahmed Ramzi, Rawan Tarek Fathi, Alaa Khogali, Faisal A. Nawaz, Rahul Kashyap, Zara Arshad

**Affiliations:** 1 Department of Orthopaedics, Malda Medical College and Hospital, Malda, India; 2 Department of Neurology, University of Minnesota, Minneapolis, Minnesota USA; 3 Faculty of Public Health, Al-Quds University, Jerusalem, Palestine; 4 Family Medicine Department, Lagos State Health Service Commission, General Hospital, Odan, Lagos, Nigeria; 5 Laboratory Of Epidemiology And Research In Health Sciences, Faculty of Medicine and Pharmacy, Sidi Mohamed Ben Abdallah University, Fez, Morocco; 6 National School of Public Health, Ministry of Health and Social Protection, Rabat, Morocco; 7 School of Medicine, David Tvildiani Medical University, Tbilisi, Georgia; 8 Department of Pediatrics, Bolan Medical College, Quetta, Balochistan Pakistan; 9 College Of Medicine, Alfaisal University, Riyadh, Saudi Arabia; 10 Institute of Medicine, Tribhuvan University, Kathmandu, Nepal; 11 Department of Internal Medicine, Trinity Health Oakland Hospital/Wayne State University, Pontiac, Michigan USA; 12 Department of Neurosurgery, Northwestern University, Chicago, Illinois United States; 13 Department of ENT, Malda Medical College and Hospital, Malda, India; 14 Alberto Hurtado Faculty Of Medicine, Universidad Peruana Cayetano Heredia, Lima, Peru; 15 Faculty Of Medicine, Alexandria University, Alexandria, Egypt; 16 Faculty Of Medicine, University Of Tripoli, Tripoli, Libya; 17 Faculty Of Medicine, Riga Stradins University, Riga, Latvia; 18 Department Of Internal Medicine, Udayapur District Hospital, Udayapur, Nepal; 19 Department of Psychiatry, Amanuel Mental Specialised Hospital, Addis Ababa, Ethiopia; 20 College of Physiotherapy and Occupational Therapy, Institute of Neurosciences, Kolkata, India; 21 Department of Neurosurgery, Armed Forces Medical College, Pune, India; 22 Clinical Skills Department of St. George’s University, True Blue, West Indies Grenada; 23 Department of Internal Medicine, Shadan Institute of Medical Sciences, Hyderabad, India; 24 College of Medicine, Mohammed Bin Rashid University of Medicine and Health Sciences, Dubai Health, Dubai, United Arab Emirates; 25 Department of Infectious Disease, Rochester General Hospital, Rochester, New York United States; 26 Department of Anaesthesiology, West China Hospital, West China Medical School, Sichuan University, Chengdu, China; 27 European Student Think Tank, Public Health And Policy Working Group, Amsterdam, Netherlands; 28 Faculty of Medicine, Mansoura University, Mansoura, Egypt; 29 Department of Gastroenterology, Nasser City Institute Hospital, Ministry of Health, Cairo, Egypt; 30 Faculty of Medicine, University of Medical Sciences and Technology, Khartoum, Sudan; 31 Emirates Health Services, Al Amal Psychiatric Hospital, Dubai, United Arab Emirates; 32 Department of Research, WellSpan Health, York, PA United States; 33 Department of Internal Medicine, Shifa International Hospital, Islamabad, Pakistan

**Keywords:** Female, Women in medicine, Doctors, Medical students, Marriage, Cultural constructs, Marital pressures, Global

## Abstract

**Background:**

Female medical students and doctors (women in medicine) face systemic gender biases, cultural expectations, and marital pressures that are linked to their career trajectories, leadership opportunities, and well-being. This study explores these barriers globally, assessing their connection to gender equity, career advancement, and retention in medicine.

**Objectives:**

To evaluate how marital and cultural pressures relate to the career progression and emotional well-being of women in medicine.

**Methods:**

A cross-sectional online survey using convenience and snowball sampling was disseminated globally through the Hub-and-Spokes model between November 2023 and December 2023. Responses (*N* = 3,031) from 53 countries were analysed in SPSS after consolidating Likert scales into three categories.

**Results:**

Most respondents (78.7%, *N* = 2386) reported that female doctors face greater societal expectations to marry, and 72.4% (*N* = 2195) noted that these expectations correspond with career choices. Marriage-related pressure from family or friends was reported by 61.5% (*N* = 1865). Over half agreed that women are expected to prioritise child-rearing (51.2%, *N* = 1552) or work part-time (52.7%, *N* = 1597). These pressures correlated with high stress (72.9%), guilt or self-blame (68.6%), and burnout (70.3%). Reported coping strategies included open communication with partners (84.6%), boundary setting (76.7%), and family or mentor support systems (72%).

**Conclusion:**

Societal and cultural pressures remain closely linked to the professional and personal challenges of women in medicine. Targeted policy reforms and supportive workplace structures are needed to promote gender equity and well-being in medicine.

**Supplementary Information:**

The online version contains supplementary material available at 10.1186/s12909-025-08519-3.

## Introduction

In a 2019 report, the Association of American Medical Colleges (AAMC) reported an increased number of women joining medical schools [1]. Similar trends have also been seen worldwide: in Singapore, applications from women for the National University of Singapore’s medical course outnumbered the school’s quota [2], while in the United Kingdom, a greater number of women than men were graduating as doctors [3]. Figures from the Organisation for Economic Cooperation and Development (OECD) also demonstrate that between 1990 and 2015, the share of female doctors in member countries went up from 29% to 46% [4].

Despite the increasing representation, significant gender disparities persist in leadership positions. Women make up approximately two-thirds of the world’s health and social care workforce, while men still hold 69% of chief executive positions and 80% of chairman positions internationally [5]. A mere 20% of organizations have gender parity on their boards, with women being most highly represented in allied health professions like nursing and midwifery [6]. Women in academic medicine have similarly been regarded as having a “leaky pipeline” career path, where they drop out of the profession before reaching senior or leadership roles due to issues like gender bias, lack of mentorship, childcare responsibilities, and work–life conflict [7, 8]. 

The 18 to 34 year-old global average age of marriage frequently corresponds with the training and early-career phases of medical doctors [9, 10]. Societal pressures to marry at this age may impede women’s professional development [11]. Research has indicated that marriage and childbearing correlate with inhibited career development among female doctors [12]. Nevertheless, much of the literature that exists centres on mid-career doctors, without a comprehensive investigation of how marital expectations and cultural norms influence women’s experiences throughout medical school and early practice [13–15]. 

With these long-standing inequalities and the lack of evidence at the early-career phase, a global view is essential to understand how medical training intersects with cultural and social expectations. The IMPACT-F study fills this niche by investigating how marital pressures and cultural constructs influence the career progression and emotional well-being of women in medicine across 53 countries.

## Materials and methods

### Study design

The study was a cross-sectional observational study that employed an online survey to capture responses from international participants. The study period was from 24th November 2023 to 22nd December 2023. This study, adhering to the format of previous global surveys under the Global Remote Research Scholars Program (GRRSP), was disseminated with a “hub and spoke model.” In this model, the hub core team conceived the study and created a questionnaire, which was disseminated by researchers of various nationalities to their country-specific networks (spokes) [16, 17]. 

### The survey questionnaire and validation

The survey questionnaire (Supplementary File 1) was created on Google Forms through a step-by-step process. First, a detailed search of open-access literature was carried out to identify pre-existing questionnaires related to our field of study. Since no existing questionnaire was available, a tailored survey was designed. The final survey contained 34 questions answered on a 5-point Likert scale (Strongly Disagree, Disagree, Neutral, Agree, Strongly Agree). The questionnaire included four thematic domains consisting of 25 items in total: perceptions of societal and marital expectations (6 items), impact on career (5 items), emotional impact (7 items), and coping strategies and support mechanisms (7 items).

Validation of questionnaires was done through a two-stage process: internal validation through guidance from mentors to avoid leading questions and external validation through field experts. External validators’ feedback resulted in some changes, such as the inclusion of ‘Societal perception of age of marriage/having children’ as a possible societal pressure on women in medicine. Some other changes involved updating ‘Divorced’ to ‘Divorced/Separated’. Initially restricted to Southeast Asia and the Middle East, the survey was later scaled up globally on the advice of experts. Upon testing and validation, the pilot testing was completed with the lead co-authors to look for any inconsistencies and to identify the average time taken to fill out the survey. The final survey was rolled out on November 24, 2023.

### Sample size estimation and study population

Assuming availability to 1% of the world’s roughly 5 million registered doctors and medical students (50,000) and a 20% frequency of the research condition, a minimum of 3,000 replies was aimed for in order to have a 1.6% margin of error. The sample size formula and its variables are explained in detail in Supplementary File 1.

The population for the study consisted of medical students and health workers of any sex worldwide; however, most were female or male, 1% were gender variant, non-conforming, transgender, or did not want to disclose (see Table 1).

**Table 1:** Demographic characteristics of survey respondents (*N* = 3031)

	Variables	Frequency	Percentage (%)
Gender	Female	1996	65.9
	Male	1002	33.1
	Other (Gender Variant/Non-Confirming/Transgender/Prefer Not to Disclose)	33	1.0
Age	18–25	1864	61.5
	26–35	846	27.9
	36–45	195	6.4
	46–55	93	3.1
	56+	33	1.1
Ethnicity	South Asian	1086	35.8
	Black - African	420	13.9
	West Asian and North African	401	13.2
	Other/Prefer not to disclose	336	11.1
	White Caucasian/Slavic/Ukrainian/Russian	328	10.8
	Hispanic/Latino	132	4.4
	Mixed Race	113	3.7
	East Asian	92	3
	Central Asian	48	1.6
	US (American Indian/Alaska Native/Asian American/Black or African American/Native Hawaiian/Pacific Islander)/others	38	1.3
	Southeast Asian	37	1.2
Marital status	Unmarried	2313	76.3
	Married	602	19.9
	Divorced/Separated/Widowed	68	2.2
	Others	48	1.6
Work position	Medical student	1589	52.4
	Resident/junior resident/MD/MS in training/Medical Officer/House Officer/Fellow/DM/MCH in Training	740	24.4
	Attending Physician/Consultant/Faculty/Administration/Medical Director	316	10.4
	Intern	244	8.1
	Others (Research Physician, Tutor)	142	4.7
Years of clinical experience	< 1	1738	57.3
	1–5	753	24.8
	6–10	267	8.8
	11–20	181	6
	≥ 21	92	3
Medical Specialty	None	1500	49.5
	Medical	629	20.8
	Others	515	17
	Surgical	387	12.8
Countries by Income Level	Lower-middle-income country	1431	47.2
	Upper-middle-income country	765	25.2
	High-income country	513	16.9
	Low-income country	322	10.6

### Recruitment procedure

The sampling was conducted employing the ‘snowballing’ method [18]. The dissemination was coordinated by a core team of seven members from five countries through weekly video calls and a special WhatsApp group. All the team members used their professional networks, sending the survey through direct messages, email, and WhatsApp. The country leads, and representatives acted as champions, promoting diverse respondent representation in their respective networks. Tracking was made easy by monitoring responses on a daily basis and grouping them by countries. To further increase participation from outside the originally represented nations, a digital survey banner was designed and posted on Facebook and LinkedIn, reaching a globalized international healthcare community.

### Data cleaning and statistical analysis

Information gathered via Google Forms was first tabulated and cleaned up in Microsoft Excel. Responses on Likert scales were reduced to a 3-point scale (Agree, Neutral, Disagree) to enhance interpretability as well as simplify comparison between gender and regional subgroups. This classification also reduced data sparsity among the underrepresented response alternatives, making it possible to conduct more powerful statistical testing within the large multi-country sample. We recognize that this strategy might have limited granularity and possibly concealed fine-grained variation in participant response, which is identified as a study limitation. Countries were categorized according to income level based on standard income categories as: low, lower-middle, upper-middle, and high-income [19]. We contrasted female and male respondents’ answers on perceptions, attitudes, experiences, and practices concerning pressures for marriage in society, as indicated in Table 2. Statistical tests, such as p-value determination, were conducted solely for these comparisons to ascertain the significance of the differences observed. All the descriptive statistics are presented in the form “% (N = )” to provide uniformity and ease of understanding in the text, tables, and figures. Responses from participants self-identifying as gender variant, non-conforming, transgender, or who did not want to report their gender were reported separately in Table 7 of Supplementary File 2. Statistical analysis was conducted using IBM SPSS version 28.

## Results

### Survey response representation

**Fig. 1:**
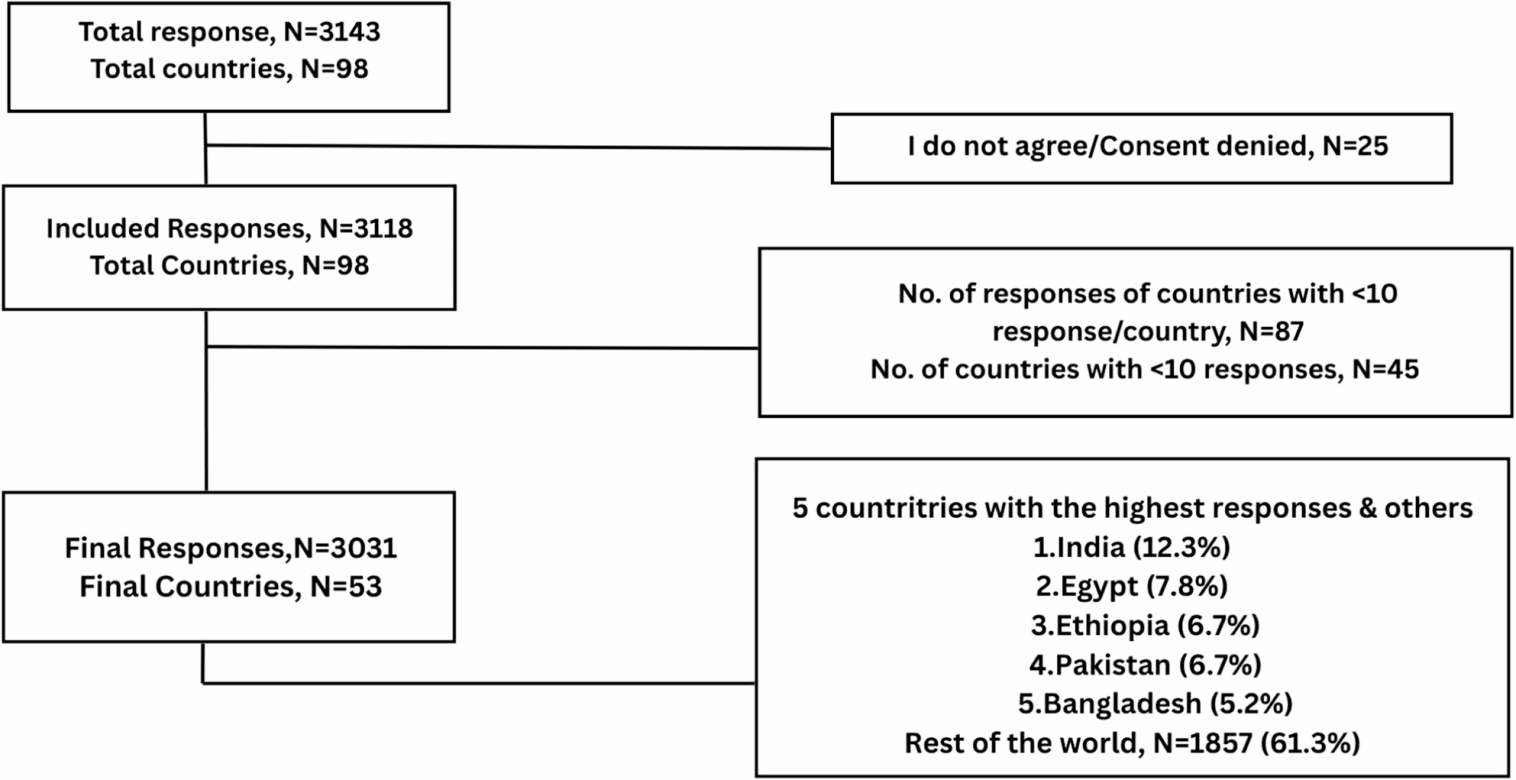
Cumulative survey response

Over the course of four weeks, 3,143 responses were received from doctors and medical students across 98 countries. Twenty-five participants (0.8%) declined consent to participate, and their responses were excluded. A total of 3,031 valid responses (96.4%) from 53 countries were included in the final analysis. Among the five countries contributing the most responses, India contributed the highest number (*N* = 374, 12.3%), followed by Egypt (*N* = 235, 7.8%), Ethiopia (*N* = 204, 6.7%), Pakistan (*N* = 203, 6.7%), and Bangladesh (*N* = 158, 5.2%). The remaining 48 countries comprised the rest of the 1857 (61.3%) responses (Fig. 1).

### Demographic characteristics

Most of the participants (*N* = 1996, 65.9%) were female, whereas 33.1% were male (Table 1). Of these, over 60% of the participants were aged 18–25 years, whereas nearly 28% of the respondents were aged 26–35 years (*N* = 846). In terms of geography, South Asians were the most prominent group (*N* = 1086, 35.8%), followed by Black-Africans (*N* = 420, 13.9%), and West Asian and North African (*N* = 401, 13.2%). Approximately 76% (*N* = 2313) of the participants were unmarried, followed by married (*N* = 602, 19.9%).

Work roles included medical students being the highest group (*N* = 1589, 52.4%), followed by Resident/Junior Resident/MD/MS in training/Medical Officer/House Officer/Fellow/DM/ MCH in training (*N* = 740, 24.4%).

Most of the respondents had < 1 year of clinical experience (*N* = 1738, 57.3%), followed by 1–5 years of clinical experience.

### Perceptions, attitudes, experiences and practices

Most respondents (78.7%, *N* = 2386) agreed that social pressure to marry was experienced more by female doctors than by male doctors. Among the total sample, 72.4% (*N* = 2195) responded that the pressure was identified as being part of the line of work of women in medicine. Also, 72.1% (*N* = 2185) agreed that pressure from society towards marriage can have detrimental effects on the mental well-being of women in medicine.

**Fig. 2:**
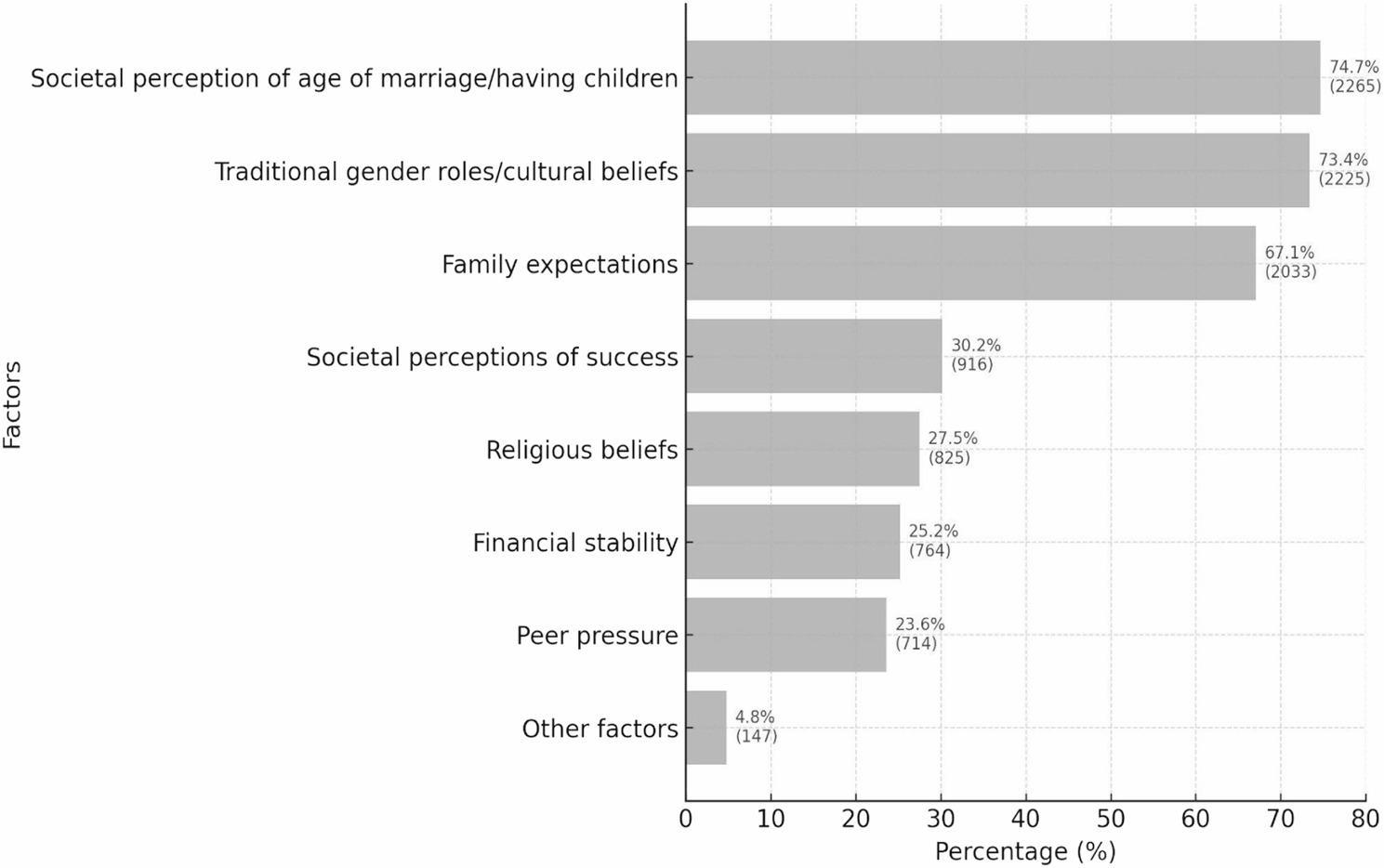
Top factors contributing towards societal pressure towards marriage for female medical students and doctors

For the eight reasons behind social pressure towards marriage, the three most prominent were perception of age of marriage/children by society (74.7%, *N* = 2265), cultural or traditional gender roles (73.4%, *N* = 2225), and expectations of family (67.1%, *N* = 2033) (Fig. 2).

Likewise, in relation to the proposition that women in medicine have ongoing pressure from family or friends to marry in society, 53.6% (*N* = 537) of males agreed, 28.4% (*N* = 285) were neutral, and 18.0% (*N* = 180) disagreed (Table 2). Female participants showed greater agreement with 65.6% (*N* = 1309) agreeing, 19.7% (*N* = 393) neutral, and 14.7% (*N* = 294) disagreeing (Table 2). Chi-square tests supported a strong relationship between female gender and answer to each statement (*p* < 0.001).

**Table 2:** Survey results on perceptions, attitudes, and experiences regarding societal pressures for marriage among women in medicine

Statement	Frequencies and Percentages			*p*-value
	Female (*N* = 1,996)	Male (*N* = 1,002)	Others (*N* = 33)	
1. Societal pressure for marriage is greater for female doctors than for their male counterparts.	Agree: 1,664 (83.4%) Neutral: 208 (10.4%) Disagree: 124 (6.2%)	Agree: 701 (70.0%) Neutral: 184 (18.4%) Disagree: 117 (11.7%)	Agree: 21 (63.6%) Neutral: 5 (15.2%) Disagree: 7 (21.2%)	< 0.001
2. Societal pressure for marriage plays a major role in shaping the career paths of women in medicine.	Agree: 1,519 (76.1%) Neutral: 258 (12.9%) Disagree: 219 (11.0%)	Agree: 657 (65.6%) Neutral: 196 (19.6%) Disagree: 149 (14.9%)	Agree: 19 (57.6%) Neutral: 6 (18.2%) Disagree: 8 (24.2%)	< 0.001
3. Women in medicine experience constant societal pressure to get married from family or friends.	Agree: 1,309 (65.6%) Neutral: 393 (19.7%) Disagree: 294 (14.7%)	Agree**:** 537 (53.6%) Neutral: 285 (28.4%) Disagree: 180 (18.0%)	Agree: 19 (57.6%) Neutral: 4 (12.1%) Disagree: 10 (30.3%)	< 0.001
4. Societal pressure for marriage can negatively affect the mental health of women in medicine.	Agree: 1,504 (75.4%) Neutral: 305 (15.3%) Disagree: 187 (9.4%)	Agree: 657 (65.6%) Neutral: 215 (21.5%) Disagree: 130 (13.0%)	Agree: 24 (72.1%) Neutral: 5 (15.2%) Disagree: 4 (12.1%)	< 0.001
5. While I have not personally faced societal pressure for marriage, my fellow women in medicine have.	Agree: 1,168 (58.5%) Neutral: 520 (26.1%) Disagree: 308 (15.4%)	Agree: 470 (46.9%) Neutral: 327 (32.6%) Disagree: 205 (20.5%)	Agree: 19 (57.6%) Neutral: 7 (21.2%) Disagree: 7 (21.2%)	< 0.001

### Associations with career advancement

Results in Table 3 show that 56.6% (*N* = 1715) of respondents held the view that women professionals in the medical sector should marry earlier than men, regardless of whether or not they had career goals.

Similarly, 52.7% (*N* = 1597) agreed that working part-time to fulfil family and domestic duties is a requirement for women in medicine, and 51.2% (*N* = 1552) said that putting childbearing ahead of career development is generally expected of women in medicine.

**Table 3:** Perceived career expectations for women in medicine

In my opinion, women in medicine are expected to:	Response	Frequency	Percentage (%)
1. Marry at a younger age compared to males, irrespective of their career goals.	Agree	1715	56.6
	Neutral	529	17.5
	Disagree	787	26
2. Prioritise having children over their careers.	Agree	1552	51.2
	Neutral	699	23.1
	Disagree	780	25.7
3. Work part-time to take care of family and household responsibilities.	Agree	1597	52.7
	Neutral	714	23.5
	Disagree	720	23.7
4. Prioritise their spouse/partner’s career before their own career.	Agree	1405	46.3
	Neutral	669	22.1
	Disagree	957	31.6
5. Take a break from their career once they are married.	Agree	1237	40.8
	Neutral	683	22.5
	Disagree	1111	36.6

### Associations with emotional well-being

As indicated by Table 4, 72.9% (*N* = 2211) of the respondents recognized that women in medicine feel stress in juggling career and marital responsibilities, and 70.3% (*N* = 2132) identified burnout as a result of the imbalance.

**Table 4:** Perceived emotional impact of marital pressures and cultural constructs on women in medicine

In my opinion, women in medicine are often:	Response	Frequency	Percentage (%)
1. “Worried” that delaying marriage for their career may negatively impact their personal life.	Agree	2064	68.1
	Neutral	568	18.7
	Disagree	399	13.2
2. “Stressed” from juggling career and marital commitments	Agree	2211	72.9
	Neutral	560	18.5
	Disagree	260	8.6
3. Dealing with “guilt or self-blame” for not being able to balance family and career	Agree	2078	68.6
	Neutral	632	20.9
	Disagree	321	10.6
4. Dissatisfied with their “career choice” due to their marital commitments	Agree	1430	47.2
	Neutral	923	30.5
	Disagree	678	22.4
5. Dissatisfied with their “career performance” due to their marital commitments	Agree	1529	50.4
	Neutral	891	29.4
	Disagree	611	20.2
6. Experience “strain” in relationships with spouse/partner because of the demands of their medical career	Agree	1910	63
	Neutral	764	25.2
	Disagree	357	11.8
7.“Burnout” from an imbalance between career and marital commitments	Agree	2132	70.3
	Neutral	588	19.4
	Disagree	311	10.3

In addition, 68.6% (*N* = 2078) agreed that women in medicine usually feel guilt or self-blame since they cannot effectively balance family and work-related duties.

### Coping mechanisms

The findings from the survey presented in Table 5 represent a high degree of consensus, with 84.6% (*N* = 2564) endorsing open communication with one’s spouse or partner regarding career aspirations and priorities. Defining clear boundaries between work life and personal life was supported by 76.7% (*N* = 2326) of participants. Further, 72% (*N* = 2182) endorsed creating a system of family or in-law support to facilitate the balance of career and personal duties.

**Table 5:** Coping mechanisms for women in medicine as perceived by survey respondents

In my opinion, these coping strategies for female doctors can help with societal pressures for marriage:	Response	Frequency	Percentage (%)
1. Open communication with their spouse/partner about their career goals and priorities.	Agree	2564	84.6
	Neutral	286	9.4
	Disagree	181	6
2. Implementing flexible work hours and opting for part-time practice.	Agree	2172	71.7
	Neutral	559	18.4
	Disagree	300	9.9
3. Seeking support from mentors or senior colleagues.	Agree	2123	70
	Neutral	637	21
	Disagree	271	8.9
4. Setting clear boundaries between personal and professional life.	Agree	2326	76.7
	Neutral	487	16.1
	Disagree	218	7.2
5. Opting/Opted for a life partner with a similar profession.	Agree	1479	48.8
	Neutral	939	31
	Disagree	613	20.2
6. Setting up a family/parents/in-law support system.	Agree	2182	72
	Neutral	615	20.3
	Disagree	234	7.7
7. Professional help or counselling.	Agree	2033	67.1
	Neutral	722	23.8
	Disagree	276	9.1

## Discussion

In our cross-sectional worldwide survey, we discovered that women in medicine are seen to experience much more societal pressure regarding marriage and family life than their male peers. The social expectations, which are based on historical gender roles, not only influence career paths but also lead to stress, guilt, and burnout in women in medicine.

Building on these overall perceptions, gender comparisons revealed notable differences between responses from women and men. Female respondents uniformly reported greater agreement than their male counterparts, who were more likely to hold neutral or disagreeing positions. Such a trend is consistent with well-established differences between the experience and perception of gender inequality by profession. A vast majority of respondents (78.7%) admitted that females in medicine have higher social pressures to marry, a view consistent with more general studies on gender norms within the academy and medicine. The label “leftover ladies” applied in some cultural circles to shame single, professional women demonstrates how such beliefs are prevalent [20]. 

In addition to societal expectations surrounding marriage, respondents also highlighted pressures related to motherhood and work structure. Previous studies show that women are under greater social pressure to marry and have a perceived ‘shelf life,’ while men are given more latitude in negotiating this phase [21]. In this survey, 83.4% of female participants agreed that this pressure is higher for female doctors compared with their male counterparts, than did 70% of male participants (*p* < 0.001). Likewise, 76.1% of women affirmed that there is significant pressure from society in influencing professional careers, in contrast to 65.6% of men (*p* < 0.001). These findings concur with other previous research [22, 23] that discovered women consistently feel more gender inequality at whatever stage of career or type of institution.

Over half of the survey respondents (51.2%) agreed that women doctors usually put children first in their careers, and 52.7% agreed that they might be expected to work part-time to meet family commitments. These results corroborate with Bakkensen et al. [24] who also found that female doctors commonly modify their careers to meet family demands: 28.8% took prolonged leave, 24.8% altered specialities, and almost half cut working hours or turned down promotions. A few (4.3%) left medicine altogether. Buddeberg-Fischer et al. [25] also reported that female doctors, especially those who had children, had lower rates of employment and professional accomplishment, although overall job satisfaction was the same for both men and women. Doctors who had children did have much lower satisfaction, highlighting the manner in which family obligations, and not just gender, contribute to career dissatisfaction.

These findings varied across cultural and regional contexts, with respondents from collectivist societies reporting heightened pressures. Most notably, South Asian, Middle Eastern, and African respondents collectively represented the bulk of our sample, areas where collectivist cultural values and traditional gender roles continue to have significant influence. It is likely that in these regions, family expectations and social norms exert a larger influence on personal decision-making, frequently at the expense of personal freedom [26, 27]. Research from collectivist settings like Bangladesh has indicated that individuals are more likely to be subject to family and societal expectations in making important life decisions like career or marriage, as these decisions might be considered an extension of family identity and not personal desire [28]. It is likely these dynamics can account for why the respondents from these regions reported more frequently experiencing strong cultural and marital pressures influencing their working and personal lives.

Participants also firmly agreed that women in medicine feel stress, guilt, and burnout as they balance career and family roles. A review by Karakcheyeva et al. [29] highlighted that women’s multiple social roles are a risk factor for mental health problems as a result of the burden to achieve both in the workplace and at home. The Global Gender Gap Report 2021 further documented that women with children reported higher stress and reduced productivity during the COVID-19 pandemic, with 54% experiencing lower productivity compared to 46% of men with children [30]. This reflects the “double shift” phenomenon, where women manage both formal employment and caregiving responsibilities, contributing to chronic stress and burnout [31]. 

A study among Nigerian medical doctors indicated that marital status, childbearing, and cadre of medical practice were highly related to the more prevalent stress among the subjects of the study [32]. More extensive system-level reviews have been undertaken that emphasise the importance of workforce planning, management change, and equitable policies in advancing equity in healthcare systems, as noted by El Arnaout et al. [33].

Our results show that open communication, mentorship, boundary setting, and institutional support are the most supported coping strategies among women in medicine. Parallel concerns have been underlined by Shellock et al. [34] who, in qualitative research among interdisciplinary marine scientists, found mentorship, peer support, and inclusive leadership to be essential facilitators empowering women to overcome professional obstacles and build resilience in male-dominated academic environments. Their “glass obstacle course” model demonstrates how shared institutional and interpersonal support can overcome systemic obstacles and boost women’s leadership and well-being. IMPACT-F most centrally spotlights societal and cultural pressures influencing women’s lives in medicine, and it could be difficult to directly map these sweeping equity efforts onto the particular issues we saw. However, these illustrations do offer valuable frameworks and best practices that future efforts can draw on. Our results on female medical professionals’ stress, burnout, and role conflict are consistent with international evidence: in 2020, 51% of female doctors were reported to be suffering from burnout, as opposed to 36% of men, a 42% greater prevalence, some studies indicating up to 20–60% higher rates for women [35, 36]. 

Taken together, this research reiterates that cultural and societal pressures are still exerting great career and emotional pressures on female medical professionals internationally. Correcting these disparities calls for wide-ranging institutional intervention and not just depending on personal coping mechanisms. Policies facilitating flexible training routes and the universalization of part-time or non-linear career paths are necessary to ensure that such opportunities do not impede professional progress. The elevated rates of stress and burnout serve to emphasize the necessity for low-cost, confidential, and stigma-free mental health care that addresses the gender-specific issues experienced by women in medicine. Lastly, gender-sensitivity and equity education integrated into medical school curricula can help eliminate biases at the early stages of professional training. With these system-level changes, the medical profession can make positive strides toward gender equity and long-term health of its entire workforce.

### Strengths

Firstly, with a robust response of over 3,000 participants, the study ensures reliability, allowing for meaningful subgroup analyses (Cronbach’s alpha = 0.8). Secondly, the study had responses from both males and females, thus allowing gender balance in the perspective. Thirdly, responses from culturally diverse regions provide a broader global perspective, making the argument of the study stronger and the findings more generalizable. Finally, the questionnaire was subjected to internal and external validation to ascertain its reliability, clarity, and cultural fit for various respondents.

### Limitations

Although we have made an effort to report data from a representative and diverse population, the study has a number of limitations. We completed the survey only in English, which might have limited access for participants for whom English was not their first language and could have introduced measurement bias. We recognize the possibility of selection bias at many levels.

First, reliance on voluntary feedback via professional networks could have drawn in participants with more entrenched opinions on the subject. Second, the snowball sampling method led to overrepresentation of a few countries with more vibrant respondent networks (e.g., Egypt, India) and underrepresentation of others. This skewing may have influenced the perspectives captured and, as a result, limits the generalizability of the findings.

While the sample size of around 3,000 respondents increases reliability, it is not necessarily representative of the global context or experiences of all female healthcare workers. Regional comparison (e.g., South Asia vs. MENA) would have been more culturally rich, but in this dataset, such an analysis was not possible. Region-stratified sampling should be used in future studies to mitigate bias and enhance representativeness.

While Likert-scale answers were combined descriptively for simplicity, more sophisticated statistical methods like ordinal or logistic regression might provide more insightful exploration of stress, burnout, and role conflict-related factors. Likewise, subgroup analysis by income level of country, marital status, and geographic region was precluded by uneven information distribution in the subgroups; further investigation could be made by future studies using stratified sampling or multivariate regression.

Lastly, the lack of open-ended questions limited the capacity to apprehend qualitative aspects of marital and cultural pressures that can enhance prospective investigations.

### Future directions

Our results highlight a significant concern: existing work and marriage regulations do not serve the needs of female medical professionals, and change is required. Further studies could focus on promoting work-life balance models that transcend traditional gender expectations, as well as examining the reasoning behind respondents’ differing opinions on how gender norms have changed for the medical profession. In addition, there are a few topics for future research, such as auditing initiatives to measure female medical professionals’ achievements in career development and job satisfaction, examining how work policies and initiatives are advancing gender equality within the profession, and conducting longitudinal studies to examine career trajectories in female medical professionals across time.

## Conclusion

Our analysis brings to light challenges and pressures faced by women medical professionals while dealing with gender, marriage, and cultural expectations as they traverse their medical careers. Women doctors were more likely to indicate prioritizing marital duties, working part-time, and reporting high stress and burnout levels while trying to balance professional and household duties. Our findings highlight the need for institutional changes that tackle the structural and cultural underpinnings associated with gender imbalance in medicine.

For medical education and workforce policy stakeholders, a number of strategies are worth considering. These include incorporating gender-sensitivity and equity modules in undergraduate and postgraduate courses, instituting formal mentorship and sponsorship programs for the career development of women, and formulating flexible training and parental-leave policies that balance family duties with professional advancement. Taken together, these reforms contribute to the vision of inclusive systems where women in medicine are able to flourish professionally and personally.

## Supplementary Information

Supplementary Material 1.

Supplementary Material 2.

## Data Availability

Data is available by contacting the corresponding author.

## Abbreviations

N: Total number of responses recorded within each category
GRRSP: Global Remote Research Scholars Program
