# Supplementary material for: Influence of marital pressures and cultural constructs on females (IMPACT-F): a global study of female medical students and doctors
Source: BMC Med Educ. 2026 Jan 3;26:182. doi: 10.1186/s12909-025-08519-3 (PMC12866102; doi:10.1186/s12909-025-08519-3)
Supplement: Supplementary file 1 — Supplementary Material 1. [file 12909_2025_8519_MOESM1_ESM.docx]

**The Survey Questionnaire:**

**Influence of Marital Pressures And Cultural Constructs on Females (IMPACT-F): A Global Study of Medical Students and Physicians**

This survey aims to gather valuable insights into how societal expectations surrounding marriage affect the experiences of female medical students and female physicians globally.

This survey is designed to collect responses from individuals of all genders. Your participation is entirely voluntary. The survey is anonymous, meaning your responses cannot be linked back to you. It consists of 5 sections and will take approximately **5–7 minutes** to complete.

We deeply appreciate your time and input on this important matter.

If you have any questions or suggestions, please feel free to reach out to:
 **Dr. Umme S. Faisal** – [ummesfaisal97@gmail.com](mailto:ummesfaisal97@gmail.com)
 **Dr. Zara Arshad** – zara_arshad@live.com
 **Dr. Rahul Kashyap** – grrspofficial@gmail.com

**Consent to Participate:**To participate in the survey, we kindly request your consent indicating that you understand the purpose of this study and are aware of your rights as a participant. By checking the box below, you agree to participate in the survey voluntarily. All information you provide will be kept confidential and used solely for research purposes. The results of this study may be published in a peer-reviewed journal; however, no personally identifiable information will be disclosed.
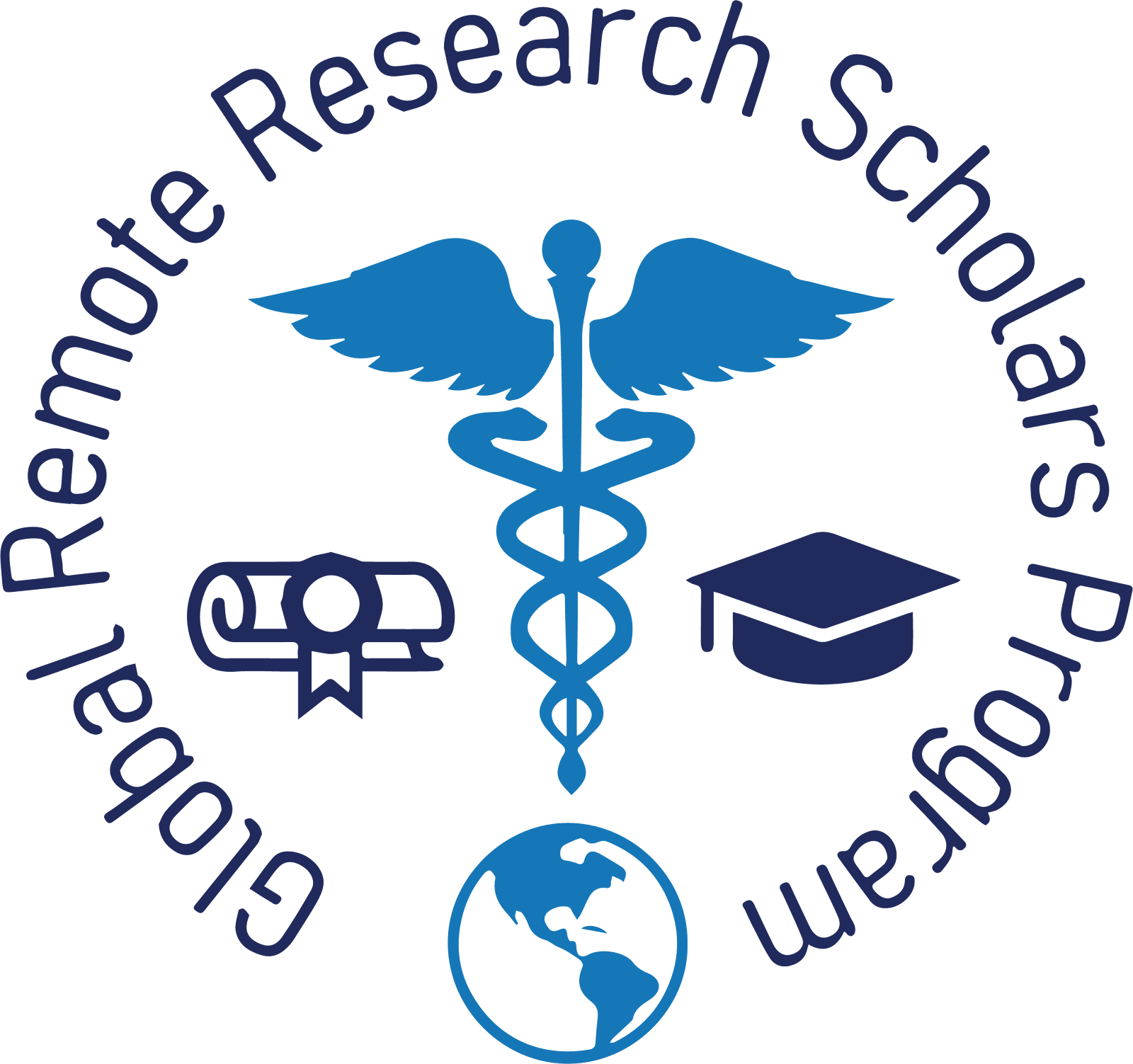


**Mark only one square:**

☐ I agree

☐ I do not agree

**Section A: Perceptions, Attitudes, Experiences/Practices**

**Instructions:**Please indicate your level of agreement with the following statements using a five-point Likert scale.
**(Strongly Disagree / Disagree / Neutral / Agree / Strongly Agree)**

| **I believe that:** | **Strongly Disagree** | **Disagree** | **Neutral** | **Agree** | **Strongly Agree** |
| --- | --- | --- | --- | --- | --- |
| **1. Societal pressure for marriage is more for female doctors than their male counterparts.** | ☐ | ☐ | ☐ | ☐ | ☐ |
| **2. Societal pressure for marriage plays a major role in shaping the career paths of female medical students and female doctors.** | ☐ | ☐ | ☐ | ☐ | ☐ |
| **3. Female medical students and female doctors experience constant societal pressure to get married from family or friends.** | ☐ | ☐ | ☐ | ☐ | ☐ |
| **4. Societal pressure for marriage can impact the mental health of female medical students and female doctors negatively.** | ☐ | ☐ | ☐ | ☐ | ☐ |
| **5. Not me, but my fellow female medical students and female doctors have faced societal pressure for marriage.** | ☐ | ☐ | ☐ | ☐ | ☐ |

**In my opinion, these are the top factors that contribute to societal pressure towards marriage for female medical students and female physicians:***(Please check all that apply)*

**Factors Influencing Personal Decisions:**

☐ Traditional gender roles / cultural beliefs

☐ Family expectations

☐ Peer pressure

☐ Societal perception of age of marriage / having children

☐ Societal perceptions of success

☐ Financial stability

☐ Religious beliefs

☐ Others (please specify): ____________

### **Section B: Career Progression and Marriage**

**Instructions:**Please indicate your level of agreement with the following statements using a five-point Likert scale.
**(Strongly Disagree / Disagree / Neutral / Agree / Strongly Agree)**

| **In my opinion, female medical students and female doctors are expected to:** | **Strongly Disagree** | **Disagree** | **Neutral** | **Agree** | **Strongly Agree** |
| --- | --- | --- | --- | --- | --- |
| **1. Marry at a younger age compared to males, irrespective of their career goals.** | ☐ | ☐ | ☐ | ☐ | ☐ |
| **2. Prioritize having children over their careers.** | ☐ | ☐ | ☐ | ☐ | ☐ |
| **3. Work part-time to take care of family and household responsibilities.** | ☐ | ☐ | ☐ | ☐ | ☐ |
| **4. Prioritize their spouse/partner’s career before their own career.** | ☐ | ☐ | ☐ | ☐ | ☐ |
| **5. Take a break from their career once they are married.** | ☐ | ☐ | ☐ | ☐ | ☐ |

### **Section C: Emotional Well-being**

**Instructions:**Please indicate your level of agreement with the following statements using a five-point Likert scale.
**(Strongly Disagree / Disagree / Neutral / Agree / Strongly Agree)**

| **In my opinion, female medical students and female doctors are often:** | **Strongly Disagree** | **Disagree** | **Neutral** | **Agree** | **Strongly Agree** |
| --- | --- | --- | --- | --- | --- |
| **1."Worried" that delaying marriage for their career may negatively impact their personal life.** | ☐ | ☐ | ☐ | ☐ | ☐ |
| **2."Stressed" from juggling career and marital commitments.** | ☐ | ☐ | ☐ | ☐ | ☐ |
| **3. Dealing with "guilt or self-blame" for not being able to balance family and career.** | ☐ | ☐ | ☐ | ☐ | ☐ |
| **4. Dissatisfied with their "career choice" due to their marital commitments.** | ☐ | ☐ | ☐ | ☐ | ☐ |
| **5. Dissatisfied with their "career performance" due to their marital commitments.** | ☐ | ☐ | ☐ | ☐ | ☐ |
| **6. Experiencing "strain" in relationships with spouse/partner because of the demands of their medical career.** | ☐ | ☐ | ☐ | ☐ | ☐ |
| **7. Experiencing "burnout" from an imbalance between career and marital commitments.** | ☐ | ☐ | ☐ | ☐ | ☐ |

### **Section D: Coping Strategies**

**Instructions:**Please express your level of agreement with the following statements using a five-point Likert scale.
**Strongly Disagree / Disagree / Neutral / Agree / Strongly Agree**

| **In my opinion, the following coping strategies can help female physicians manage societal pressures related to marriage:** | **Strongly Disagree** | **Disagree** | **Neutral** | **Agree** | **Strongly Agree** |
| --- | --- | --- | --- | --- | --- |
| **1. Open communication with their spouse or partner about career goals and priorities.** | ☐ | ☐ | ☐ | ☐ | ☐ |
| **2. Implementing flexible work hours or opting for part-time practice.** | ☐ | ☐ | ☐ | ☐ | ☐ |
| **3. Seeking support from mentors or senior colleagues.** | ☐ | ☐ | ☐ | ☐ | ☐ |
| **4. Setting clear boundaries between personal and professional life.** | ☐ | ☐ | ☐ | ☐ | ☐ |
| **5. Choosing a life partner from a similar professional background.** | ☐ | ☐ | ☐ | ☐ | ☐ |
| **6. Establishing a supportive system involving family, parents, or in-laws.** | ☐ | ☐ | ☐ | ☐ | ☐ |
| **7. Seeking professional help or counselling.** | ☐ | ☐ | ☐ | ☐ | ☐ |

### **Section G: Demographics**

**1. What is your age?** *(Select one)* ☐ 18–25
 ☐ 26–35
 ☐ 36–45
 ☐ 46–55
 ☐ 56–65
 ☐ 65+

**2. What is your marital status?** *(Select one)* ☐ Unmarried
 ☐ Married
 ☐ Divorced/Separated
 ☐ Widowed
 ☐ Others

**3. What is your current country of residence?** *(Select one)* ☐ [Dropdown list of countries, e.g., India, United States, etc.]

**4. What is your ethnicity?** *(Select one)* ☐ US-American Indian or Alaska Native
 ☐ US-Asian American
 ☐ US-Black or African American
 ☐ US-Native Hawaiian or Other Pacific Islander
 ☐ White Caucasian
 ☐ Black - Africa
 ☐ Central Asian
 ☐ East Asian
 ☐ South Asian
 ☐ West Asian and North Africa
 ☐ Southeast Asian
 ☐ Spanish/Hispanic/Latino
 ☐ Slavic/Ukrainian/Russians
 ☐ Mixed Race
 ☐ Other / Prefer not to disclose

**5. What is your state/province?** [Write-in text box]

**6. What is your gender?** *(Select one)* ☐ Male
 ☐ Female
 ☐ Transgender
 ☐ Gender Variant / Non-conforming
 ☐ Other / Prefer not to disclose

**7. What is your current role?** *(Select one)* ☐ Attending Physician / Consultant / Faculty
 ☐ Fellow / DM / MCh in training
 ☐ Resident / Junior Resident / MD / MS in training
 ☐ Medical Officer / House Officer
 ☐ Research Physician
 ☐ Administrator / Medical Director
 ☐ Medical Student
 ☐ Intern
 ☐ Other

**8. How many total years of experience do you have in the healthcare field (count after medical school graduation)?** *(Select one)* ☐ 0 (for medical students)
 ☐ <1
 ☐ 1–2
 ☐ 2–5
 ☐ 6–10
 ☐ 11–20
 ☐ 21–30
 ☐ >30

**9. What is your medical specialty?** *(Select one)* ☐ Anesthesiology
 ☐ Biochemistry / Microbiology
 ☐ Critical Care Medicine
 ☐ Dermatology
 ☐ Family Medicine
 ☐ Internal Medicine
 ☐ Neurology
 ☐ Obstetrics and Gynecology
 ☐ Ophthalmology
 ☐ Orthopedic Surgery
 ☐ Otorhinolaryngology (ENT)
 ☐ Pathology
 ☐ Pediatrics
 ☐ Plastic Surgery
 ☐ Psychiatry
 ☐ Public Health and General Preventive Medicine
 ☐ Radiology
 ☐ Research
 ☐ Surgery
 ☐ Other
 ☐ None

**Sample Size Estimation**

The sample size was estimated using the CheckMarket online calculator, assuming access to approximately 1% of the world’s 5 million registered doctors and medical students (≈ 50,000) and an expected prevalence of 20%. The margin of error was set at 1.6%, providing a minimum target of 3,000 valid responses.

The detailed sample size formula used was:


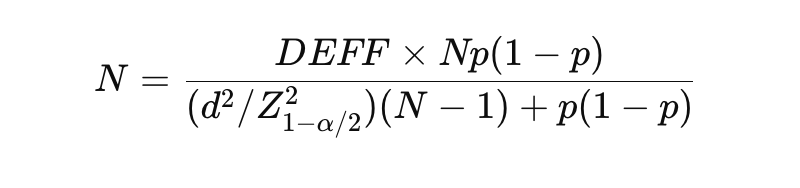


Where:

- N = total target population
- p = estimated proportion of the population exhibiting the outcome of interest
- d = margin of error (expressed as a decimal)
- DEFF = design effect
- Z 1-α/2 = standard normal deviate corresponding to the desired confidence level (1.96 for 95% CI)

This formula provided a minimum required sample of ~3,000 participants, ensuring adequate statistical precision across multiple subgroups and regions.
