# Supplementary material for: Influence of marital pressures and cultural constructs on females (IMPACT-F): a global study of female medical students and doctors
Source: BMC Med Educ. 2026 Jan 3;26:182. doi: 10.1186/s12909-025-08519-3 (PMC12866102; doi:10.1186/s12909-025-08519-3)
Supplement: Supplementary file 2 — Supplementary Material 2. [file 12909_2025_8519_MOESM2_ESM.docx]

**Additional tables and figues:**

**Table 6: Country Demographics of Survey Respondents (53 countries)**

| **Country** | **Count (N=3031)** | **Percentage (%)** |
| --- | --- | --- |
| **India** | 374 | 12.3 |
| **Egypt** | 235 | 7.8 |
| **Ethiopia** | 204 | 6.7 |
| **Pakistan** | 203 | 6.7 |
| **Bangladesh** | 158 | 5.2 |
| **Mauritius** | 141 | 4.7 |
| **Morocco** | 135 | 4.5 |
| **Palestine** | 134 | 4.4 |
| **Libya** | 116 | 3.8 |
| **Peru** | 104 | 3.4 |
| **Nepal** | 98 | 3.2 |
| **United Arab Emirates** | 98 | 3.2 |
| **United States Of America** | 61 | 2 |
| **Georgia** | 56 | 1.8 |
| **Sudan** | 53 | 1.7 |
| **Germany** | 50 | 1.6 |
| **Kazakhstan** | 50 | 1.6 |
| **Italy** | 49 | 1.6 |
| **Latvia** | 42 | 1.4 |
| **Cyprus** | 38 | 1.3 |
| **Lebanon** | 36 | 1.2 |
| **United Kingdom** | 33 | 1.1 |
| **China** | 29 | 1 |
| **Japan** | 29 | 1 |
| **Burundi** | 28 | 0.9 |
| **Syria** | 28 | 0.9 |
| **Nigeria** | 26 | 0.9 |
| **Australia** | 25 | 0.8 |
| **Brazil** | 25 | 0.8 |
| **Jordan** | 24 | 0.8 |
| **Malaysia** | 24 | 0.8 |
| **Namibia** | 22 | 0.7 |
| **Kenya** | 21 | 0.7 |
| **Sri Lanka** | 21 | 0.7 |
| **Tanzania** | 21 | 0.7 |
| **Grenada** | 19 | 0.6 |
| **Oman** | 18 | 0.6 |
| **Saudi Arabia** | 18 | 0.6 |
| **Afghanistan** | 16 | 0.5 |
| **Vietnam** | 16 | 0.5 |
| **Switzerland** | 15 | 0.5 |
| **Canada** | 13 | 0.4 |
| **Korea south** | 13 | 0.4 |
| **Ukraine** | 13 | 0.4 |
| **Indonesia** | 12 | 0.4 |
| **Russia** | 12 | 0.4 |
| **Greece** | 11 | 0.4 |
| **Iran** | 11 | 0.4 |
| **Iraq** | 11 | 0.4 |
| **Mongolia** | 11 | 0.4 |
| **Rwanda** | 11 | 0.4 |
| **Argentina** | 10 | 0.3 |
| **Austria** | 10 | 0.3 |

| **Table 7: Response of Other (Gender Variant/Non-Confirming/Transgender/Prefer Not to Disclose) with respect to Perceptions, Attitudes, and Experiences and Practices regarding societal pressures for marriage among Female Medical Students and Doctors.** | | | | |
| --- | --- | --- | --- | --- |
| **I believe that:** | **Other (Gender Variant/Non-Confirming/Transgender/Prefer Not to Disclose)**   **(N,%)** | | | **p-value** |
|  | **Agree** | **Neutral** | **Disagree** |  |
| **1. The societal pressure for marriage is more for female doctors than their male counterparts** | 21  (63.6) | 5  (15.2) | 7  (21.2) | <0.001 |
| **2. The societal pressure for marriage plays a major role in shaping the career paths of women in medicine.** | 19  (57.6) | 6  (18.2) | 8  (24.2) | <0.001 |
| **3. Women in medicine experience constant societal pressure to get married from family or friends.** | 19  (57.6) | 4  (12.1) | 10  (30.3) | <0.001 |
| **4. The societal pressure for marriage can impact the mental health of women in medicine negatively.** | 24  (72.1) | 5  (15.2) | 4  (12.1) | <0.001 |
| **5. Not me but my fellow women in medicine have faced societal pressure for marriage.** | 19  (57.6) | 7  (21.2) | 7  (21.2) | <0.001 |

**Figure 3: Figure showing the representative countries which were divided into Low-Income, Lower-Middle Income and Upper-Middle Income and High-Income countries.**
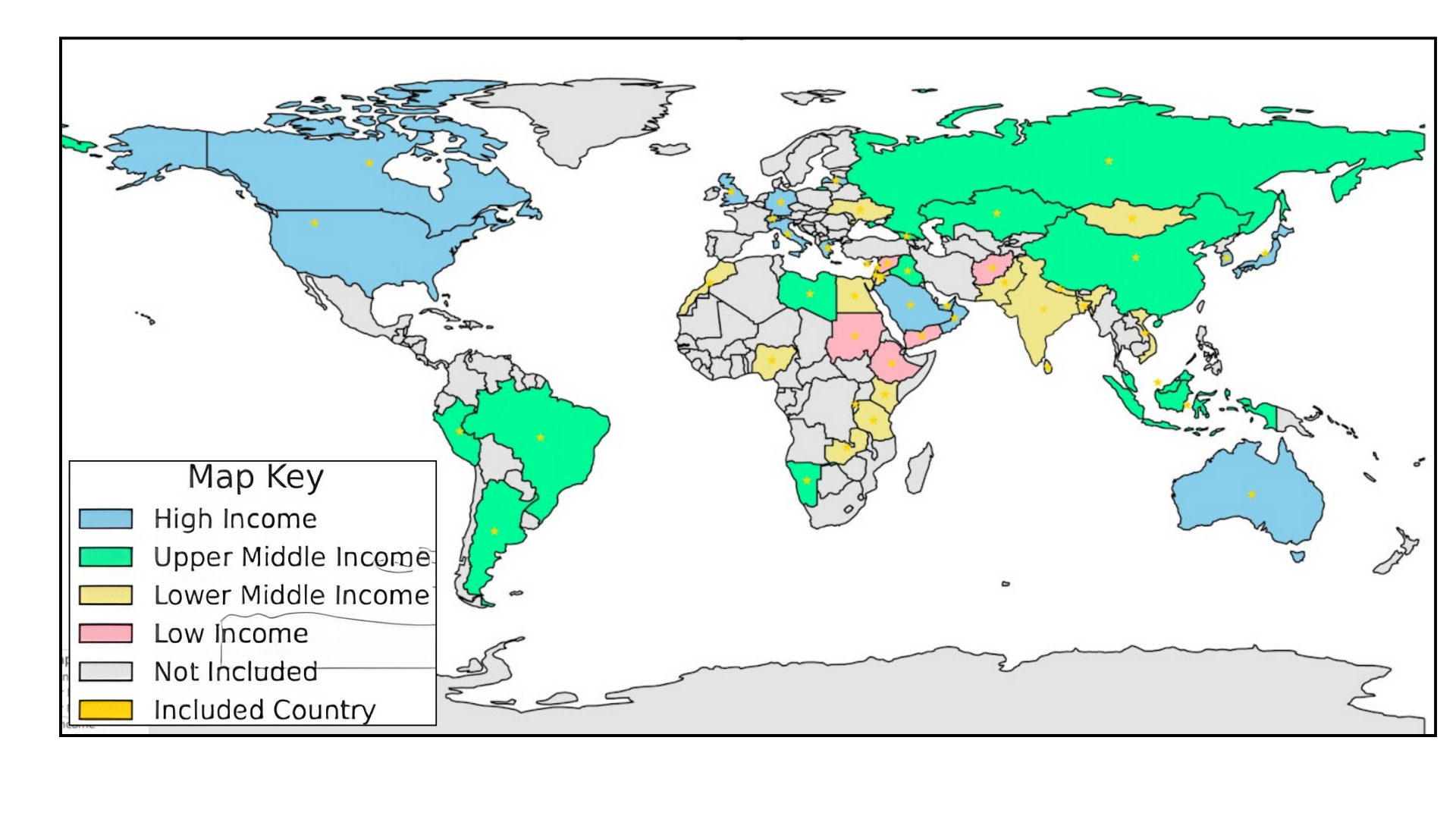


#

#

#

#

# **Country-wise Collaborative Co-Author List (53 countries)**

| **Country Name** | **Count (N=3031)** | **Collaborators** |
| --- | --- | --- |
| **India** | 374 | Umme Summaiya Faisal,Kamal Kishan Akash, Rahul Kashyap,  Umme Habiba Faisal,  Jaiprakash Gurav, Romana Riyaz,Sharanya Ezhilarasan Santhi |
| **Egypt** | 235 | Israa Ahmed Qutob, Rawan Tarek Fathi  Ahmed Ramzi Shiha |
| **Ethiopia** | 204 | Mikias Lewoyehu Wondie |
| **Pakistan** | 203 | Awranoos Ahadi, Umme Habiba Faisal,Muhammad Wajeeh Nazar,Sanaullah |
| **Bangladesh** | 158 | Ashmita Yadav |
| **Mauritius** | 141 | Abhishek Kashyap,Kriti Kamna,  Lakshitha |
| **Morocco** | 135 | Majid Omari |
| **Palestine** | 134 | Sally Shayeb  Israa Ahmed Qutob |
| **Libya** | 116 | Naiela Almansouri,  Israa Ahmed Qutob |
| **Peru** | 104 | Norma Nicole Gamarra Valverde |
| **Nepal** | 98 | Aron Shrestha,Niguma Rayamajhi |
| **United Arab Emirates** | 98 | Sameer Asim Khan, Ahmedyar Hasan |
| **United States Of America** | 61 | Rahul Kashyap, Sharanya Ezhilarasan Santhi, Roopali Dahiya |
| **Georgia** | 56 | Nino Dekanoidze  Joanna Lee |
| **Sudan** | 53 | Alaa Khogali |
| **Germany** | 50 | Darja Golubeva,Alen Sam Saji |
| **Kazakhstan** | 50 | Kriti Kamna |
| **Italy** | 49 | Rahul Kashyap, Roopali Dahiya, Salim Surani |
| **Latvia** | 42 | Nino Dekanoidze, Joanna Lee |
| **Cyprus** | 38 | Lama ZenEddin,Israa Qutob, Priyadarshini Bhattacharjee, Alaa Khogali |
| **Lebanon** | 36 | Darja Golubeva |
| **United Kingdom** | 33 | Kriti Kamna, Abhishek Kashyap |
| **China** | 29 | Alen Sam Saji, Alaa Khogali |
| **Japan** | 29 | Alma Sato, Darja Golubeva |
| **Burundi** | 28 | Alma Sato |
| **Syria** | 28 | Israa Qutob |
| **Nigeria** | 26 | Azeezat Oyewande,  Priyadarshani Bhattacharjee, Malavika Jayan |
| **Australia** | 25 | Alen Sam Saji, Aron Shrestha |
| **Brazil** | 25 | Heloísa de Souza Cavalcante, Reshon Hadmon, Alma Sato |
| **Jordan** | 24 | Israa Qutob |
| **Malaysia** | 24 | Aishah Ibrahim,Ashmita Yadav, Alen Sam Saji |
| **Namibia** | 22 | Henriette Paulus, Alaa Khogali |
| **Kenya** | 21 | Priyadarshini Bhattacharjee, Rebecca Ateino |
| **Sri Lanka** | 21 | Jaiprakash Gurav, Alen Sam Saji |
| **Tanzania** | 21 | Priyadarshini Bhattacharjee, Alaa Khogali |
| **Grenada** | 19 | Reshon Hadmon |
| **Oman** | 18 | Ahmedyar Hasan |
| **Saudi Arabia** | 18 | Maryam Asif |
| **Afghanistan** | 16 | Sanaullah |
| **Vietnam** | 16 | Aron Shrestha |
| **Switzerland** | 15 | Christos Tsagaris |
| **Canada** | 13 | Razan Adam, Abhishek Kashyap, Alaa Khogali, Roopali Dahiya |
| **Korea south** | 13 | Joanna Lee |
| **Ukraine** | 13 | Malavika Jayan |
| **Indonesia** | 12 | Alen Sam Saji |
| **Russia** | 12 | Darja Golubeva |
| **Greece** | 11 | Christos Tsagkaris |
| **Iran** | 11 | Alen Sam Saji |
| **Iraq** | 11 | Darja Golubeva |
| **Mongolia** | 11 | Aron Shrestha |
| **Rwanda** | 11 | Alaa Khogali, Emmanuel Mwizerwa Bunani |
| **Argentina** | 10 | Reshon Hadmon, Heloísa de Souza Cavalcante |
| **Austria** | 10 | Christos Tsagkaris, Darja Golubeva |
| **Total** | 3031 |  |

## 
